# Unusual location and complication of a cystic lymphangioma: A case report

**DOI:** 10.1016/j.amsu.2020.08.029

**Published:** 2020-08-29

**Authors:** Wael Rebai, Ahmed Ben Mahmoud, Yacine ouadi, Anis Haddad, Souhaieb Atri, Montasser Kacem

**Affiliations:** a Department of General Surgery A, Rabta Hospital, Tunis, Tunisia; b Faculty of Medicine of Tunis, Tunis El Manar University, Tunis, Tunisia

**Keywords:** Cystic lymphangioma, Surgery, Emergency, Acute abdomen, Case report

## Abstract

**Introduction:**

This paper reports an uncommon location and complication of a cystic lymphangioma. Few cases of infected colonic cystic lymphangioma were described in literature. Symptoms are non-specific and setting the diagnosis on radiological features remain challenging. Urgent open surgery is generally required for therapeutic and diagnostic purposes.

**Presentation of case:**

We describe a case of a young man who presented with an acute abdomen mimicking bile peritonitis, infected tumor of the colon or even a complicated hydatid disease of the liver, which is endemic in our country. CT-scan was compulsory to suspect the diagnosis of infected cystic lymphangioma but remained insufficient to rule out other more frequent diagnoses. The patient underwent an emergency open surgery consisting in a bowel resection, in whose case an infected cystic lymphangioma was barely suspected preoperatively but confirmed by the pathological examination of the specimen. No postoperative complications were noticed.

**Discussion:**

Setting the diagnosis of an infected cystic lymphangioma of the colon is tough. Many differential diagnoses are more frequently suspected and radiological examinations can be helpful. However, an emergency surgery is mandatory in order to avoid septic shock and resect the lesion, sometimes at the cost of bowel resection. Laparoscopic or endoscopic treatments are feasible but are not the standard in emergency cases.

**Conclusion:**

Acute presentation of cystic lymphangioma of the colon is very scarce and can be life-threatening leading to urgent open surgery, although endoscopic or laparoscopic treatment remain feasible. Further studies are needed to select which technique is suitable for this disorder.

## Highlights

• Cystic lymphangioma is a benign lesion.
• Colonic location of cystic lymphangiomas is scarce.
• Infected cystic lymphangiomas of the colon is rarely described in literature.
• Open Surgery is the standard treatment of cystic lymphangiomas especially in case of emergency.

## 1 Introduction

Lymphangiomas are benign lesions, most commonly seen in children. Lymphangiomas of the colon are scarce, representing less than 5% of all lymphangiomas. Although most lymphangiomas remain asymptomatic, complicated ones may lead to an emergency surgery. The final diagnosis is obtained through histological examination of the specimen. We report herein an uncommon case of a 25-year-old man who underwent urgent open surgery for an acute abdomen due to an infected cystic mass of the ascending colon related to an infected lymphangioma.

The case report has been reported in line with the SCARE criteria [1].

## 2 Case presentation

A 25-year-old man with no past medical or drug history, presented to the emergency room with an acute pain of the right upper quadrant and flank evolving for three days associated with fever. The patient reported no chronic abdominal pain or transit disorders.

Physical examination showed that the patient had fever 39 °c. The abdominal examination revealed the presence of a 10 cm - regularly shaped, soft, and fixed mass in the right hypochondria and flank. The rest of the abdomen revealed no remarkable abnormalities.

Laboratory tests showed a high c-reactive protein level (173.5mg/l) but a normal white blood cell count (7553/μl). No anemia nor hemostasis disorders were noticed. Furthermore, urine culture was normal.

Firstly, we decided to perform an abdominal ultrasound that showed a cystic formation of the right pericolic gutter with a thick wall and several septa within. The mass had close relationships with the sixth liver segment and the ascending colon. At first, we suspected a hydatid cyst of the liver because Tunisia is an hydatid endemic area. However, a malignant tumor of the colon could not be ruled out.

**Fig. 1:**
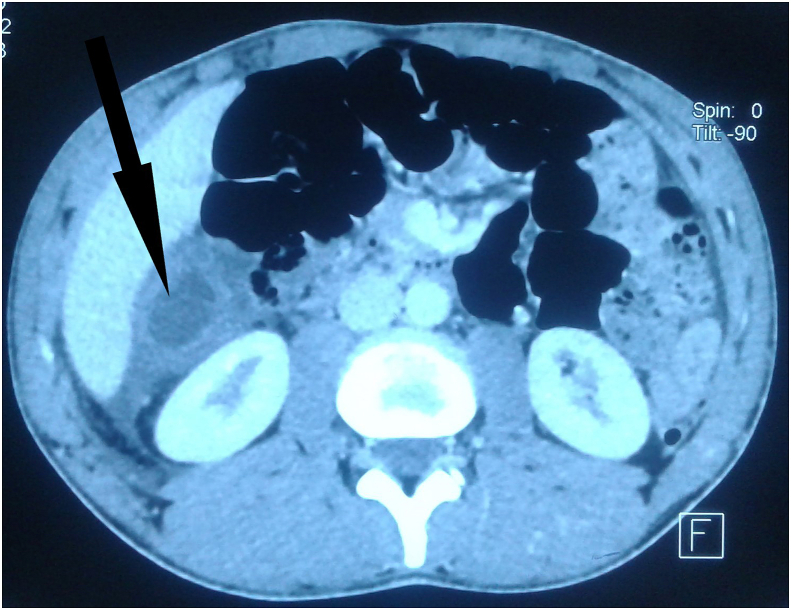
CT scan showing a cystic mass with low-enhanced thickened walls independent from the liver. The content of the cyst appears heterogonous.

**Fig. 2:**
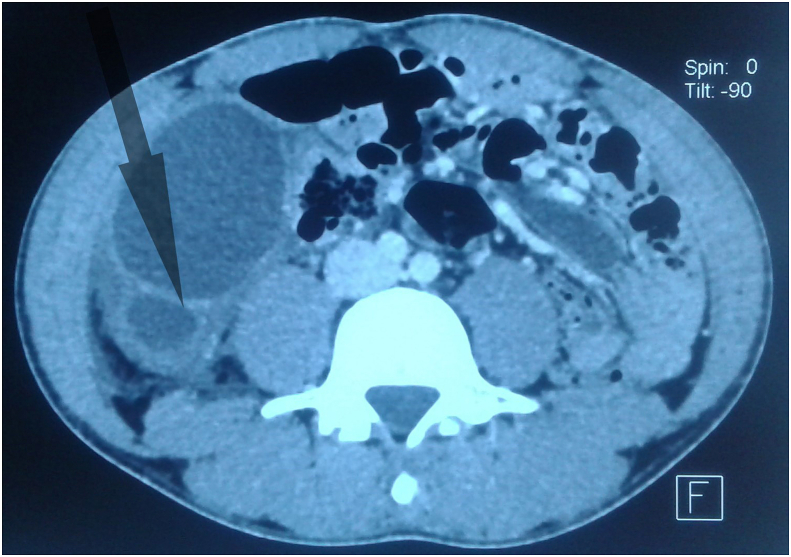
CT scan showing septa within a cystic mass with peritoneal effusion.

Therefore, we indicated an abdominal CT scan to characterize the mass and determine its exact location. It revealed a regular 10-cm- intraperitoneal cystic mass independent from the liver but repressing the ascending colon with heterogonous internal density. Moreover, the mass was multilocular edged by thick walls showing low contrast enhancement after intravenous injection. Peritoneal effusion was detected on CT scan in the pericolic gutter and douglas pouch, to boot (Fig. 1, Fig. 2).

An infected cystic lymphangioma was then suspected besides the diagnosis of intraperitoneal hydatid disease or infected malignant tumor of the colon. We decided then to perform open surgery for diagnostic and therapeutic purpose.

**Fig. 3:**
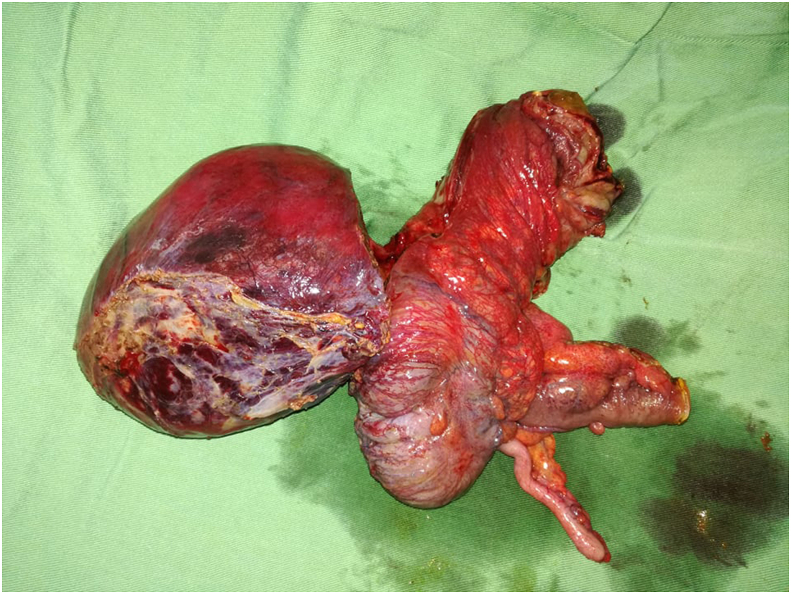
Macroscopic view of the specimen showing the cystic lymphangioma arising from the right colon with a bloody serous content.

We found a 10-cm-cystic-mass containing a serous bloody fluid in the right pericolic gutter depending from the ascending colon. The colon was not distended. No liver metastasis nor peritoneal carcinosis were noticed. An ileocolic en bloc -resection was performed with an immediate ileocolic anastomosis. (Fig. 3).

Macroscopically, the 10 –cm colonic cystic mass had many septas and contained serous fluid. It was obviously arising from the ascending colon. In addition, histopathological examination revealed dilated lymphatic and vascular components lined with attenuated endothelial cells. These findings were consistent with the diagnosis of a cystic lymphangioma. The patient's post-operative course was uneventful. He was discharged on the 5th post-operative day. A 6-months clinical and radiological follow-up showed no symptoms or recurrence with good tolerance to regular diet.

## 3 Discussion

Lymphangioma of the colon is a rare condition firstly described by Chisholm and Hillkowitz in1932 [2], and is due to a malformation of vascular or lymphatic tissues found in the submucosa, covered with normal mucosa. The most well established theory suggests that lymphangiomas arise from congenital anomalies of the lymphatic system. Subsequently they occur mainly in children [3]. Therefore adult cases are known to be very rare [4]. Lymphangiomas can occur anywhere in the body. They are usually found in the head, neck and axilla. However, those affecting the intestinal tract are scarce and most of them are located in the mesentery, the omentum and the mesocolon. Those located in the wall of the intestine are sparse and are found usually in the right half of the colon like in our case [5].

Nowadays, with the widespread use of endoscopy for preventive, diagnostic and therapeutic goals, this disorder was reported more frequently. Matsuda et al. [6]reported in 2001 a review of 279 cases of lymphangioma of the large intestine. 114 lymphangiomas within 258 were located in the right colon and in 32% of all cases reported, open colectomy was needed. Lymphangiomas are usually incidentally found by explorations done for other purposes.In some cases, the patients present with nonspecific symptoms and elective surgery is usually performed. However, complicated cystic lymphangiomas are not much described in literature and consist in lower gastrointestinal bleeding, obstruction, intussusception and protein-losing enteropathy [[7], [8], [9], [10], [11], [12]] [[7], [8], [9], [10], [11], [12]] [[7], [8], [9], [10], [11], [12]]. Therefore, it is uncommon for cystic lymphangiomas to cause severe, life-threatening abdominal symptoms in adults.What makes our case report unusual is that the chief symptom was a severe abdominal pain with signs of peritoneal irritation due to infected cystic lymphangioma. Makni et al. [13]reported one case of infected cystic lymphangioma within twenty cases of operated abdominal lymphangiomas.

Diagnosis is impossible on clinical and biological examinations alone making Imaging compulsory. The differential diagnosis includes a wide range of cystic intraabdominal lesions. The particularity of our case is that Tunisia remains a hydatid endemic area and the first diagnosis suspected for cystic mass is the intraperitoneal hydatid cyst, infected one in this case. In emergency situations US examination is the first level of imaging investigation because of its non-invasiveness, low cost, and non-use of radiation, in order to identify the lesion and to define its structural type characteristics as well as the size. However, CT scan is essential in order to obtain additional informations such as structural feature, internal and peripheral contrast enhancement, as well as loco-regional relationships and a fortiori to rule out differential diagnosis such as hydatid disease and colonic tumors. On CT, these masses show densitometry characteristics of the fluid type, regular margins, and only a peripheral contrast enhancement. For non-urgent presentations, the literature also suggests a role of MRI [14] in characterizing cystic masses. In addition, endoscopic ultrasound may be helpful. These lesions appear as anechoic, septate, and submucosal.Macroscopically, colonic lymphangiomas are classified into one of the following three types: simple (capillary), cavernous and cystic [15], the latter was found intraoperatively in our patient.

Treatment of cystic Lymphangiomas is nonconsensual and depends on its various presentations. Colonic lymphangioma can be managed by endoscopic polypectomy for lesions ranging to a maximum measurement of 2–3.5 cm [6]. Recently, laparoscopic surgery was recommended for treating lymphangiomas [16]. However, this is often difficult in emergency cases.

In our case, the choice of laparotomy was mainly driven by the fact that the patient presented with an acute abdomen and consequently, infected tumor of the colon or a hydatid cyst of the colon couldn't be excluded.

Complete resection is the standard radical treatment aiming to prevent recurrence. In fact, incomplete resection results in a 10% post-operative recurrence rate. Complete resection is essential for lymphangiomas of the intestinal tract, and usually requires bowel resection. Pathologic study is mandatory to retain the diagnosis.

## 4 Conclusion

Infected Lymphangiomas of the colon are exceptional. The diagnosis can be challenging. The management is various. An open surgery with complete resection is the standard treatment for complicated ones. Laparoscopic and endoscopic treatment hence must be considered.

## Ethical approval

There is no ethical committee in our country (Not applicable for this manuscript).

## Sources of funding

No sources of funding.

## Author contribution

All authors contributed to the study design. Wael Rebei MD (The operator), performed the surgery. Ahmed Ben Mahmoud, Yacine Ouadi and Atri Souhaieb wrote the manuscript draft. Montasser Kacem, Wael Rebei and Anis Haddad read and corrected the manuscript. All authors read and approved the final manuscript.

## Registration of research studies

It is a case report.

## Guarantor

Ahmed Ben Mahmoud.

## Consent for publication

Written informed consent was obtained from the patient for publication of this case report and any accompanying images. A copy of the written consent is available for review by the Editor-in-Chief of this journal on request.

## Provenance and peer review

Not commissioned, externally peer reviewed.

## Declaration of competing interest

All authors declare that they have no any conflicts of interest.
